# **A coaxially extruded heterogeneous core**–**shell fiber with Schwann cells and neural stem cells**

**DOI:** 10.1093/rb/rbz037

**Published:** 2019-11-07

**Authors:** Xinda Li, Dezhi Zhou, Zhizhong Jin, Hongqing Chen, Xuanzhi Wang, Xinzhi Zhang, Tao Xu

**Affiliations:** 1 Biomanufacturing and Rapid Forming Technology Key Laboratory of Beijing, Department of Mechanical Engineering, Tsinghua University, Beijing 100084, People’s Republic of China; 2 Key Laboratory for Advanced Materials Processing Technology, Ministry of Education, Department of Mechanical Engineering, Tsinghua University, Beijing 100084, People’s Republic of China; 3 Department of Neurosurgery, the First Hospital of China Medical University, Shenyang 110122, People’s Republic of China; 4 Department of Neurosurgery, Xijing Hospital, Fourth Military Medical University, Xi'an 710032, People’s Republic of China; 5 Department of Neurosurgery, the First Affiliated Hospital of Wannan Medical College, Wuhu 241001, People’s Republic of China; 6 East China Institute of Digital Medical Engineering, Shangrao 334000, People’s Republic of China; 7 Medprin Regenerative Medical Technologies Co., Ltd, Shenzhen 518102, People’s Republic of China; 8 Department of Precision Medicine and Healthcare, Tsinghua Berkeley Shenzhen Institute, Shenzhen 518055, People’s Republic of China

**Keywords:** coaxial extrusion, neural stem cell, Schwann cell

## Abstract

Cellular therapies play a critical role in the treatment of spinal cord injury (SCI). Compared with cell-seeded conduits, fully cellular grafts have more similarities with autografts, and thus might result in better regeneration effects. In this study, we fabricated Schwann cell (SC)-neural stem cell (NSC) core–shell alginate hydrogel fibers in a coaxial extrusion manner. The rat SC line RSC96 and mouse NSC line NE-4C were used in this experiment. Fully cellular components were achieved in the core portion and the relative spatial positions of these two cells partially mimic the construction of nerve fibers *in vivo*. SCs were demonstrated to express more genes of neurotrophic factors in alginate shell. Enhanced proliferation and differentiation tendency of NSCs was observed when they were co-cultured with SCs. This model has strong potential for application in SCI repair.

## Introduction

Cellular therapies are an important subject of preclinical study in the treatment of spinal cord injury (SCI) [1]. Schwann cells (SC) and neural stem cells (NSC) are two major cell types that have shown regenerative effects in rodent SCI models [2, 3]. For SC, they could result in myelination of demyelinated central nervous system (CNS) cells [4]. Moreover, SC express nerve growth factor (NGF) and brain-derived neurotrophic factor (BDNF) which could promote axon growth in SCI tissue [5, 6]. For neural stem/progenitor cells, a previous study has demonstrated that *in vitro*-expanded neural stem/progenitor cells could differentiate into neurons and participate in the repair of SCI [7]. Meanwhile, NSCs were demonstrated to secrete extensive neurotrophic factors (e.g. NGF and BDNF) and support axon growth both *in vitro* and *in vivo* [8]. However, previous researchers have realized that grafting SCs alone is still not optimal since substantial regeneration of supraspinal axons is not achieved [9, 10]. Therefore, research focusing on transplanting multiple cell types into SCI models has been performed [11]. There is also evidence that SCs can promote neural differentiation of NSCs during co-culture [12]. Based on the above reasons, we hypothesize that SCs may promote neural differentiation of NSCs in a 3D co-culture model and that this model may have potential in treatment of SCI.

Owens and colleagues have fabricated and tested a fully cellular nerve graft composed of SCs and bone marrow stem cells [13]. This graft might be similar to an autograft, which might be a section of nerve composed of a large number of cells and extracellular matrices. Meanwhile, with the aid of 3D bioprinting technology, the relative position of SCs and stem cells (designed to differentiate into neurons) could be defined. Recently, a novel core–shell cell fiber paradigm was presented by Onoe and colleagues [14]. In this paradigm, a large amount of cells and extracellular matrices are in the core portion and alginate hydrogel with certain mechanical strength is in the shell portion. Therefore, under the protection of the shell, the construction of the core is more similar to that of an autograft, mentioned above. Neural stem/progenitor cells were loaded in the core portion and mouse SCI model transplantation was performed [15]. The transplanted cells maintained the capability to proliferate and differentiate. In vitro differentiation of core NSCs was also demonstrated with induction factors [16].

Bioprinting SC-encapsulated structures have also been extensively studied [17–19]. SCs could maintain viability and proliferation capability while encapsulated in alginate-based hydrogel [17, 18]. Meanwhile, SCs could also express neurotrophic factors (e.g. NGF) in such hydrogel [20]. Furthermore, if properly modified, SCs could spread and develop dendrites in alginate-based hydrogels. The orientation of dendrites was tunable [19].

**Figure 1:**
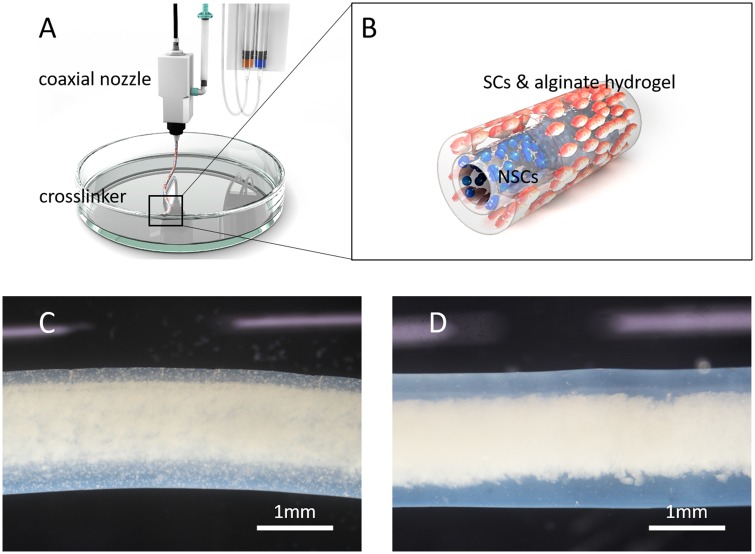
Schematic views of extrusion setup (**A**) and SC-NSC core–shell fiber (**B**). Images of SC-NSC fiber (**C**) and NSC fiber (blank) (**D**) under a stereoscope

Based on the above-mentioned progress, for the first time, we fabricated an SC-NSC co-culture model in the core–shell fiber paradigm. SCs were encapsulated in the alginate hydrogel shell and NSCs were in the core with high density (Fig. 1B). This strategy has two potential advantages: First, it partially mimics the relative spatial position of neurons and glial cells where glial cells form myelin sheaths wrapping around the axons from the neurons [21]. Second, the core portion of this construct is purely cellular which might be similar to an autograft. Rat Schwann cell line RSC96 and mouse neural stem cell line NE-4C were used in this study. A previous study of the co-culture of mouse-derived and rat-derived neural cells has shown that there was communication between these two heterogeneous cells [22]. In this brief proof-of-concept study, we hypothesize that these two cell types can communicate to a certain extent. SCs might promote neuron differentiation of NSCs as previously reported [12]. Immunostaining was performed to estimate the differentiation degree of NSCs in this co-culture model. In addition to that, a cell proliferation test was performed to verify the change in proliferation profiles of these two cell types when they were co-cultured.

## Materials and methods

### Cell culture

RSC96 cell line was purchased from American Type Culture Collection (CRL-2765) and maintained in Dulbecco’s Modified Eagle Medium (Gibco, 11965-092), supplemented with 10% fetal bovine serum (Gibco, 10099-141), 100 U/ml penicillin and 100 μg/ml streptomycin (Gibco, 15140-122). NE-4C cell line was kindly provided by Stem Cell Bank, Chinese Academy of Sciences and maintained in minimum essential medium (MEM, Gibco, 11090-081) supplemented with 10% fetal bovine serum (Gibco, 10099-141), 1% GlutaMAX^TM^ (Gibco, 35050) and 1% non-essential acids (Gibco, 11140). Both cell lines were cultured at 37°C in 5% CO_2_ and passaged every 2–3 days.

### Fiber extrusion

Sodium alginate (Sigma, A0682) powder was gamma ray sterilized and dissolved in deionized water at a concentration of 4%. RSC96s and NE-4Cs were digested when they reached 80–90% confluence. RSC96s were re-suspended in alginate solution at a concentration of 2 × 10^6^ cells ml^−1^. Meanwhile, NE-4Cs were re-suspended in MEM at a concentration of 1 × 10^8^ cells ml^−1^. For fiber extrusion, RSC96-alginate solution and NE-4C suspension were, respectively, loaded into two sterilized 10-ml syringes. The syringes were subsequently set on a two-channel syringe pump. A double coaxial device (Nayi) with a nozzle consisting of two concentric circles (0.577 and 1.469 mm; inner diameters) was utilized to extrude fibers. Briefly, RSC96-alginate solution and NE-4C suspension were connected to the outer and inner layer of the coaxial nozzle, respectively. The two components were subsequently extruded into 3% calcium chloride bath through the coaxial nozzle. Crosslinking occurred immediately when the alginate and calcium chloride contacted each other and a hydrogel shell formed. A high density of NE-4Cs was encapsulated in the core portion of the fibers (Fig. 1A and B). SC-NSC fibers were finally cultured in MEM and the medium was changed every 2–3 days. On Days 0, 3, 5, 7 and 9 post-extrusion, diameters of the fiber shell and core portion were measured under a microscope at 30 different points.

### Cell viability analysis

Cell viability was assessed with a fluorescent live/dead viability assay kit (KeyGEN, KGAF001) following the manufacturer’s instruction. Briefly, samples were immersed in staining solution containing 8 μM propidium and 2 μM Calcein-AM. After incubating for 15 min, the samples were washed three times with phosphate-buffered saline. Fluorescent images were taken with a fluorescence microscope. Live and dead cells were stained green and red by Calcein-AM and propidium. For cell viability calculations, live and dead cells were counted in at least five random sights under 100× magnification.

### Fiber appearance and cell morphology analysis

Scanning electron microscopy (SEM) was used to observe fiber appearance and cell morphology. Briefly, the samples were fixed in 2.5% glutaraldehyde for 20 min. A series of ethanol solutions with concentrations of 50, 75, 80, 90, 95 and 100% (v/v) were used to dehydrate after fixation. The samples were then dried in a vacuum drying chamber for 30 min. Images were obtained from a desk type scanning electron microscope (Phenom ProX) after sputtering.

### Shell dissolving analysis

On culture Days 1, 5 and 9, SC-NSC fiber and NSC fiber were both immersed in 55 mM sodium citrate solution and gently shaken on a rocking device. The time that alginate shell took to totally dissolve was recorded. Three replicates were conducted at each time point for each fiber.

### mRNA expression analysis

Quantitative real-time polymerase chain reaction (qRT-PCR) was performed to estimate the abundance of mRNA transcripts of samples. Briefly, RSC96s were harvested with 55 mM sodium citrate and 20 mM ethylene diamine tetraacetic acid solution and re-suspended in TRIzol (Gibco, 15596026) with repetitive pipetting. The extracted total RNA was transcribed to cDNA using ImProm-IITM Reverse Transcription System. qRT-PCR was performed with the Sequence Detection System using SYBR Green qPCR Master Mix (Invitrogen).

### Cell proliferation analysis

Cell proliferation profile was assessed with the Alamar Blue Kit (YEASEN, 40202ES76). The fibers were cut into 3 cm sections for a proliferation test. The proliferation test was implemented on Days 1, 3, 5, 7, 9, 11, 13 and 15 after samples were fabricated according to the manufacturer’s instructions. Briefly, Alamar Blue and MEM were mixed at a ratio of 1:9 to obtain a working solution. Samples were incubated with 3 ml working solution for 2 h. After the incubation, 100 μl working solution was transferred to a well of a 96-well culture plate. The optical density (OD) value of each well was read on a microplate reader at wavelengths of 570 and 600 nm. The OD values on each day were normalized to that on Day 1. Three replicates were conducted at each time point.

### Marker protein expression analysis

Immunostaining was performed to assess the expression of characteristic proteins of NSCs and neurons. Briefly, NE-4Cs were harvested from samples according to the procedures indicated in Section ‘Fiber appearance and cell morphology analysis’. Harvested cells were then seeded on a 24-well plate and incubated till they attached and developed dendrites. The cells were subsequently fixed with 4% paraformaldehyde for 30 min. Blocking solution (KeyGEN, KGIHC008) was used to block the cells for 30 min. Anti-nestin (Abcam, ab11306) and Anti beta-III tubulin (Abcam, ab18207) were diluted by primary antibody diluent (KeyGEN, KGIHC009) at manufacturer recommended concentrations. Cells were incubated with primary antibody overnight at 4°C. Secondary antibodies (Beyotime, A0568; A0562) were diluted by secondary antibody diluent (KeyGEN and KGIHC011). After aspirating the primary antibody solution, the cells were gently washed with immunostaining wash buffer (KeyGEN and KGIHC010) and incubated with corresponding secondary antibody for 1 h. Cell nuclei were finally stained with DAPI for 10 min. The fluorescent images were taken under a fluorescence microscope.

### Statistical analysis

The results are presented as mean ± standard deviation. Statistical analysis was performed using one-way analysis of variance (ANOVA) in conjugation with a student t-test. *P* < 0.05 was regarded as statistically significant. All data were analysed and presented using GraphPad Prism 5 software.

## Results

### The effects of extruding speeds on RSC96 viability and shell geometry

**Figure 2:**
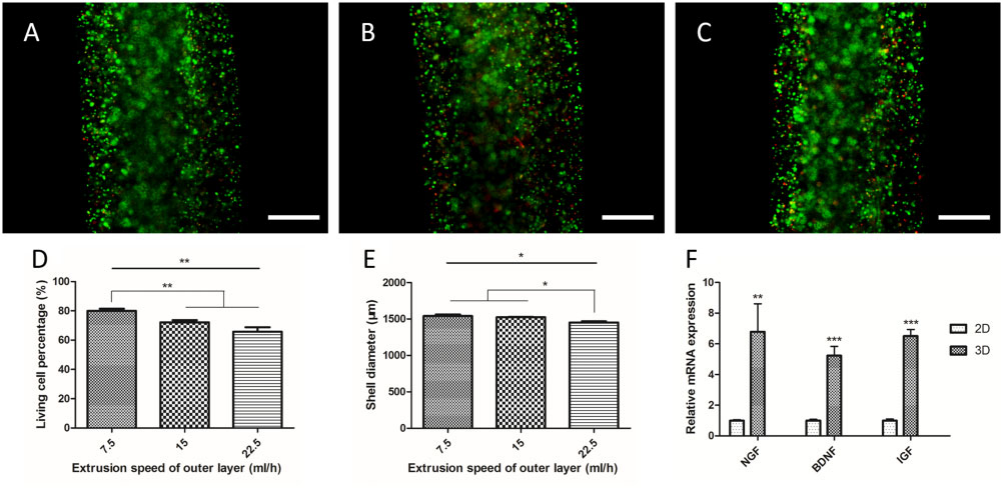
Fluorescent images of live-dead stained SC-laden shell extruded at 7.5 ml/h (**A**), 15 ml/h (**B**) and 22.5 ml/h (**C**). Cell viability (**D**) and shell diameter (**E**) at different extruding speeds (*n* = 5). (**F**) NGF, BDNF and IGF gene expression of SCs in alginate shell and petri dishes (*n* = 3)

Three outer shell layer extruding speeds were chosen to fabricate the shell portion of fibers. Briefly, the speeds were set at 7.5, 15 and 22.5 ml/h to extrude RSC96-alginate shell (Fig. 2A–C). In order to distinguish shell cells clearly, calcium chloride was extruded in the core portion in this experiment. The viability of encapsulated RSC96s and the shell layer diameters under different extruding speeds were measured. The results are shown in Fig. 2D and E. RSC96 viability under extruding speeds 7.5, 15 and 22.5 ml/h are 79.96 ± 3.02, 72.22 ± 2.76 and 65.80 ± 5.21%. There is a statistically significant difference between the 7.5  and the 15 ml/h group and no significant difference between the 15  and 22.5 ml/h group. A decrease in cell viability can be observed when increasing the extruding speed. For outer diameters of shell layer, the results are 1540.50 ± 38.7, 1524.05 ± 8.89 and 1451.71 ± 27.28 μm. In this series of results, there is a statistically significant difference between the 15 ml/h group and the 22.5 ml/h group and no significant difference between the 7.5  and 15 ml/h group. The shell diameter shows a tendency to decrease as the extruding speed increases.

### Neurotrophic factor synthesis of RSC96s in alginate

The capability of RSC96s to synthesize neurotrophic factors in alginate hydrogel was assessed by qRT-PCR. The mRNA transcript abundances of NGF, BDNF and insulin-like growth factor (IGF) of RSC96s in alginate (3D) and petri dish (2D) are shown in Fig. 2F. The mRNA transcript abundances of each factor were normalized to 2D. For NGF, the relative mRNA transcript abundances of 2D and 3D were 1.00 ± 0.05 and 6.78 ± 1.83, respectively. For BDNF, they were 1.00 ± 0.08 and 5.23 ± 0.60, respectively. Finally, for IGF, they were 1.00 ± 0.10 and 6.50 ± 0.43, respectively. For each factor, there are statistical significances between 2D RSC96s and 3D RSC96s. This result indicates that RSC96s have the potential to synthesize more neurotrophic factors in 3D alginate hydrogel. In other words, the result indicates that in this co-culture cell fiber, RSC96s in the shell portion of cell fibers may nourish the NE-4Cs in the core portion.

### Appearance of fiber shell and cell morphology

**Figure 3:**
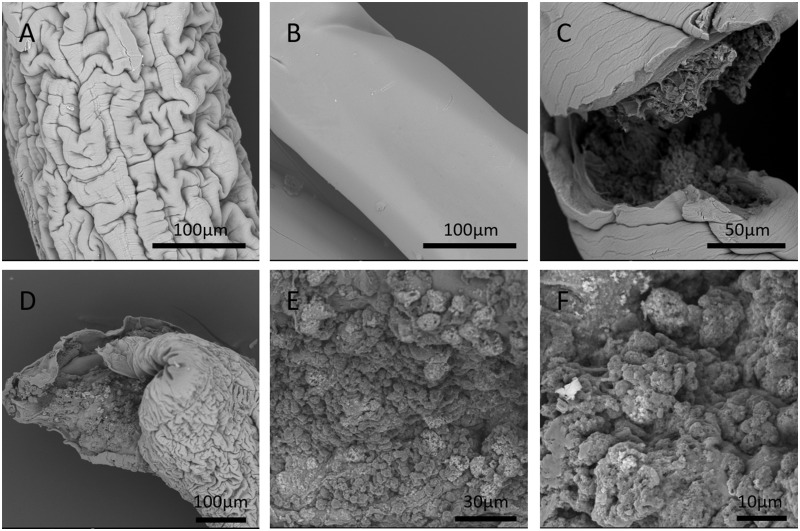
SEM images of SC-laden alginate shell (**A**) and pure alginate shell (**B**). (**C**, **D**) Fiber fractures where cells in core portion could be observed. (**E**) Pattern of NSCs in the core portion. (**F**) A more detailed view of NSCs in the core portion

**Figure 4:**
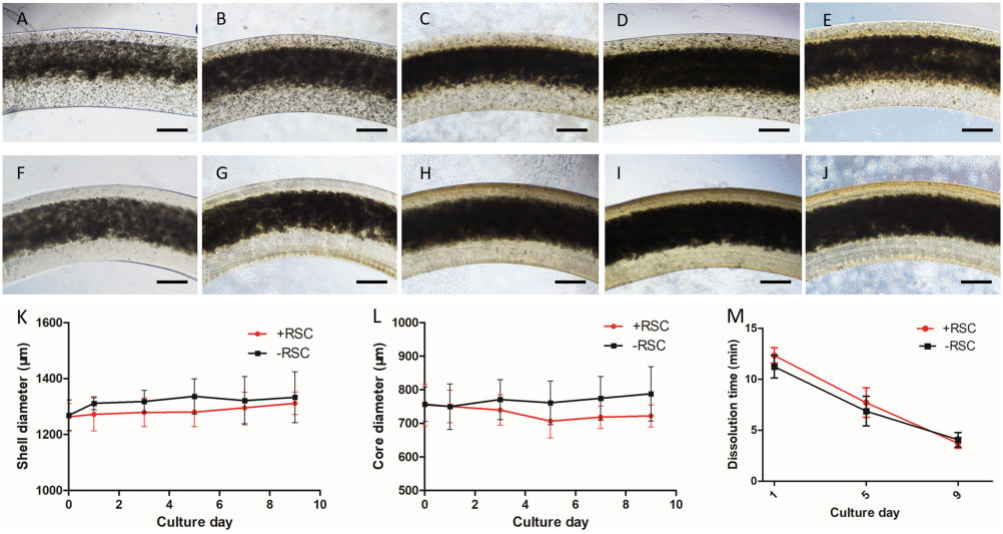
(**A**–**E**) Microscopy images of SC-NSC fiber on Day 0, 3, 5, 7, 9 post extruding. (**F**–**J**) microscopy images of NSC fiber on Day 0, 3, 5, 7, 9 post extruding. (**K**) Variation of shell diameters of SC-NSC fiber and NSC fiber (*n* = 30). (**L**) Variation of core diameters of SC-NSC fiber and NSC fiber (*n* = 30). (**M**) Dissolution time of SC-NSC fiber and NSC fiber in sodium citrate solution on Day 1, 5 and 9 post printing (*n* = 3). Scale bar: 100 μm

The SEM images of the appearances of SC-NSC and NSC fiber are shown in Fig. 3A and B. There were wrinkles on the surface of SC-NSC fiber while the surfaces of NSC fibers were smooth. From the fracture, the NSCs in the core portion could be observed (Fig. 3C and D). According to Fig. 3E and F, NSCs in the core portion grew into spheroids with a large amount of extracellular matrices or ungelled alginate permeation. Fiber appearances under a microscope are shown in Fig. 4A–J. Since the initial cell density in the core portion was rather high, NSCs proliferated and filled the core portion at around Day 5 of culturing. Both the SC-laden shell and pure alginate shell swelled during culture and the shell diameter of pure alginate shell was larger than SC-laden shell from Day 1 to the end (Fig. 4K). The core diameter of SC-NSC fiber also began to shrink from Day 1 and the difference became gradually larger (Fig. 4L). There were no statistically significant differences between both diameters of SC-NSC fiber and NSC fiber on Day 9. During culture, both shells slowly degraded and the dissolving time shortened when they were placed in alginate solving liquid (Fig. 4M). The SC-laden shell and pure alginate shell show the same trend of variation in dissolving time.

### Cell proliferation profile in core–shell fibers

**Figure 5:**
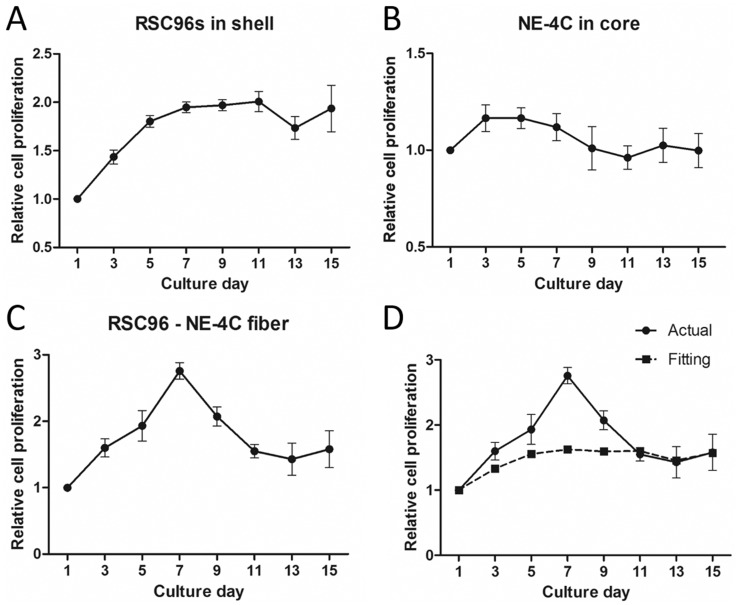
Proliferation profiles of SCs in shell (**A**), NSCs in core (**B**), SC-NSC core–shell fiber (**C**) and comparison of curve C and fitting curve of A and B (**D**) (*n* = 3)

The extruded RSC96-NE-4C fiber is shown in Fig. 4A–J. It can be observed that though NE-4Cs in the core portion were initially extruded with high density (1 × 10^8^ cells ml^−1^, Fig. 4A and F) as mentioned in Section ‘Appearance of fiber shell and cell morphology’. The cell proliferation profiles of RSC96s in the shell portion, NE-4Cs in the core portion and both cells as entireties were separately assessed. The results are shown in Fig. 5A–C. Figure 5A depicts the proliferation profile of RSC96s in the shell portion. For the initial 5 days, RSC96s proliferated rapidly and the relative OD value on Day 5 was around 1.8 times that on Day 1. From Day 5 to Day 11, the proliferation slowed down and the OD value on Day 11 reached the peak, which was around two times that on Day 1. A decrement occurred on Day 13 and the OD value subsequently increased on Day 15. Figure 5B depicts the proliferation profile of NE-4Cs in the core portion. NE-4Cs proliferated only for the initial 3 days and the relative OD value reached its peak on Day 3, which was around 1.2 times that on Day 1. From Day 3 to Day 11, the OD value kept decreasing and a remarkable decrement was observed on Day 9. On Day 11, the value fell to its low peak which was only 0.96 times that on Day 1. The OD value increased on day 13 and then decreased on Day 15. Figure 5C depicts the proliferation profile of RSC96-NE-4C core–shell fibers, in which RSC96s and NE-4Cs were co-cultured in the fiber as shown in Fig. 5. As an entirety, the OD value increased rapidly and reached its peak on Day 7, which was around 2.8 times that on Day 1. From Day 7 to Day 13, it sharply decreased and fell to 1.4 times that on Day 1. A slight increment of OD value occurred on Day 15, and the final OD value was 1.58 times that on Day 1.

Based on the linear relation between OD value and cell number in the alamar blue test, the proliferation curves of RSC96s in the shell and NE-4Cs in the core were fitted and compared with the entire proliferation curve, which is shown in Fig. 5D. Briefly, the variation of OD value caused by a 3-cm section of RSC96 shell or a 3-cm section of NE-4C core cultured for 2 h was separately measured. The normalized OD values of RSC96 shell and NE-4C core at each time point were integrated with the weighted average based on the OD value variation caused by each component. It can be observed from Fig. 5D that the entire proliferation curve of the core–shell fiber is higher than the fitted curve from Day 3 to Day 9. This result might indicate that one cell type accelerated the proliferation of the other in the co-culture cell fiber.

### Neuron differentiation of NE-4Cs in the core

**Figure 6:**
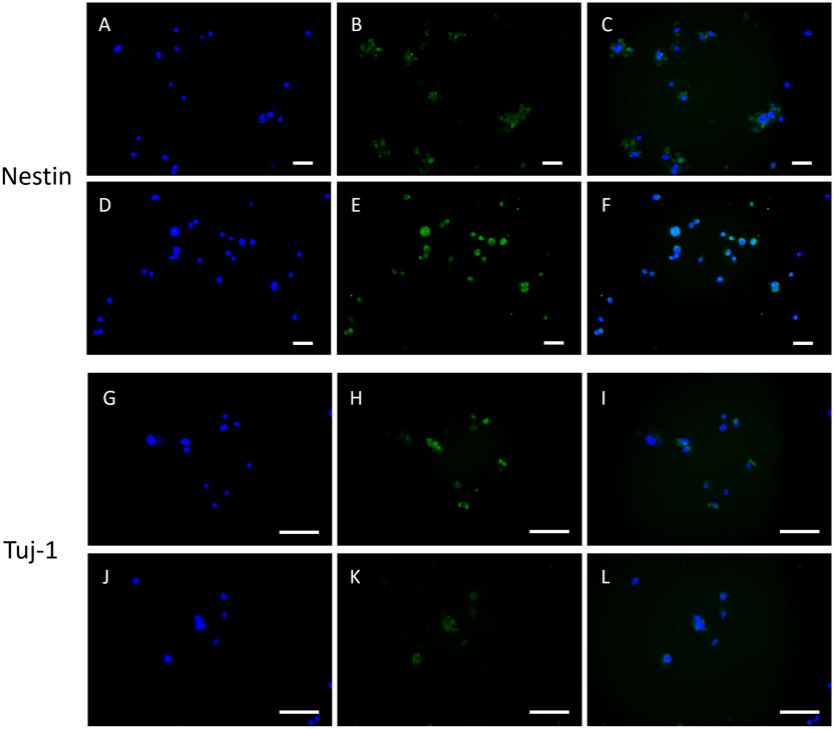
Immunostaining of nestin and tuj-1. (**A**–**C**) DAPI, nestin and merged image of NSCs from SC-NSC group. (**D**–**F**) DAPI, nestin and merged image of NSCs from NSC group. (**G**–**I**) DAPI, tuj-1 and merged image of NSCs from SC-NSC group. (**J**–**L**) DAPI, tuj-1 and merged image of NSCs from NSC group. Scale bar: 100 μm

NE-4C cells were extracted from the cell fibers on Day 7 after extrusion according to the procedures in section ‘mRNA expression analysis’. The harvested NE-4Cs were cultured in petri dishes until cells attached. Immunostaining was implemented on petri dishes. NE-4Cs were stained with nestin and beta-III tubulin (tuj-1), which are NSC marker and neuron marker, respectively. The results are shown in Fig. 6. Figure 6A–C presents nestin staining of NE-4C cells extracted from fibers with RSC96s in the shell (SC-NSC). Figure 6D–F presents nestin staining of NE-4C cells extracted from fibers without RSC96 in the shell (NSC only). It can be found from Fig. 6A–F that the fluorescence intensity of the SC group is weaker than that of the blank group. Figure 6G–I and Fig. 6J–L present the tuj-1 staining of the SC group and the blank group, respectively. It can be found that the tuj-1 fluorescence of the SC group is stronger than that of the blank group.

## Discussion

We successfully fabricated SC-NSC core–shell fibers with crosslinked alginate hydrogel as a structural support. The core portion and the shell portion were extruded simultaneously through a coaxial nozzle. The tip of the nozzle was immersed in calcium chloride crosslinker (Fig. 1A). Therefore, the SC-suspended sodium alginate solution crosslinked immediately when extruded and the NSC core portion that was extruded were wrapped in without leakage. The extruding process could be continuous, which resulted in meter-long core–shell single fiber accumulation in the crosslinker container. Therefore, there were unavoidable points of flexion along the fiber. Due to the crosslinking of the alginate, these flexion points were fixed and non-deformable. Fractures might occur at these sites when culture time was prolonged and alginate was degraded. Generally, a single fiber might break into 1–5 cm sections on around Day 7 of culturing. These sections would maintain structural integrity themselves with slight cell leakage. For this reason, a single cell fiber was cut into 3 cm sections before experiments were performed.

SC viability and fiber geometry were measured with the variation of shell extrusion speed. The impairment to SCs increases with the increase of extrusion speed. This result may be due to the increase of the shear stress. In fact, the cell viability under the minimum experimental extrusion speed (7.5 ml/h) is 79.96 ± 3.02%, which is not high. This result might be due to the extrusion setup. There was around 20 cm of perfusion tube connecting the syringe nozzle and the coaxial nozzle inlet. Since the cell-alginate solution has non-negligible viscosity, shear stress would accumulate in the tube and impair the cells. The viability of the NSCs in the core part is not presented in this manuscript since the cell density is high and the fluorescent cells could not be distinguished when live-dead staining was performed. A previous work in our group revealed that core cell viability was around 96% when extrusion was performed in this manner [23]. In the core portion, there was a pure cell suspension whose viscosity was far lower than alginate solution, resulting in lower shear stress and impairment to cells. The shell diameter decreased as the extrusion speed increased (Fig. 2E). Due to the setup, the fiber dropped into the crosslinker bath after extrusion. In this experiment, the fibers were extruded in 10 cm petri dishes which were rather shallow. Therefore, the accumulated fibers underneath the nozzle were dragged aside manually to avoid nozzle clogging. Higher extrusion speed resulted in larger material outflow per unit time. We suppose that the more frequent dragging might increase tension on the fiber and decrease the diameter.

Wrinkles were found on the surface of the SC-laden alginate shell under a scanning electron microscope (Fig. 3A) while no wrinkles were observed on pure alginate shell (Fig. 3B). Similar wrinkles could be observed on the surface of alginate dialdehyde which was due to partial oxidation [24]. Therefore, the result in this experiment might be due to the various degrees of shrinkage of SCs and alginate gel during dehydration or due to certain reactions between alginate and SC excretions. NSCs in the core portion were observed to grow into spheroids under scanning electron microscope (Fig. 3E and F). However, with an optical microscope, we found that NSCs grew into fiber-like shapes during culture (Fig. 4A–J). Therefore, we concluded that NSCs in the core portion aggregated and grew into spheroids during initial culture. As culture time prolonged, these spheroids got bigger and connected to each other with loose cell junctions. There were also extracellular matrices secreted by NSCs and ungelled alginate in the gaps of cell spheroids. While the fibers were dehydrated and dried, due to different shrinking percentages of cell spheroids, extracellular matrices and alginate gels, NSCs in the core portion presented their spheroid morphology in which the cells connected tightly.

The SCs were demonstrated to express more neurotrophic factor RNA in alginate shells than in 2D petri dishes (Fig. 2F). On one hand, neurotrophic factors such as NGF and BDNF have beneficial effects on neurons in both peripheral and CNS [25, 26]. On the other hand, co-culture of SCs and stem cells could result in neural differentiation of stem cells [27–29], to which SC secretion partially contributes [29]. Since the initial cell density in the core portion of the fiber was high (Fig. 4A and F), NSCs were not able to maintain long-time proliferation without other nutrition support (Fig. 5B). The proliferation curves of single SC shell and single NSC core were weighted averaged where the weight was a variation of OD value caused by these two constructs. The weighted averaged curve was compared with the proliferation curve of SC-NSC fibers (Fig. 5D). The SC-NSC curve was higher than the fitted curve from Day 3 to Day 9. We hypothesize that the shell SCs might have a nourishment effect on core NSCs. Furthermore, NSCs were demonstrated to secrete neurotrophic factors [8], which might nourish the shell SCs as well. Neural differentiation of NSCs was found to be stronger in the SC-NSC group than in the NSC group. This result might indicate that SCs have an induction effect on NSCs. It has been demonstrated that NSCs transplanted into SCI remained largely undifferentiated [3]. Therefore, neural induction can be performed in advance of transplantation intending to make the donor cells contribute to cellular replacement [30]. We believe that if neuron induction of NSCs could be achieved in this model, the heterogeneous fibers would be directly transplanted into SCI models without *in vitro* induction. Moreover, Onoe and colleagues presented in their study that the diameter of the NSC core portion showed 0.36–0.46 folds decrease when neural induction was performed, indicating neural differentiation [16]. In this study, the core diameter of SC-NSC fiber was also slightly smaller than that of NSC fiber during 9-day culturing (Fig. 4L). Though there was no statistically significant difference between these two groups, the result might indicate that the NSCs co-cultured with SCs had a higher tendency to differentiate, compared with pure NSC fiber, where the NSCs might contribute more to cellular replacement in possible transplantations. In fact, co-culture of SCs and NSCs in core–shell fiber was only demonstrated to enhance the tendency for NSCs to differentiate since no neuron-like cells were observed when the NSCs were digested on Day 7 of culture.

One of the major concerns is that heterogeneous cells were used. Therefore, previous studies were investigated and we found that interactions of heterogeneous cells did exist in co-culture [22, 29, 31]. Watabe and colleagues found that the mouse glial cell line-derived neurotrophic factor cDNA coding region had 97% homology with the corresponding rat cDNA sequence [31]. This result might provide supporting evidence for our study. As a proof-of-concept study, we preliminarily found nourishment and differentiation effects of this SC-NSC core–shell fiber model. There are still some deficiencies to be addressed. First, primary cells should be used for a better estimation of regeneration. Second, a small quantity of extracellular matrix should be added for cells to maintain normal morphologies, which might be critical for good function. We might address these in our future works.

## Conclusion

SC-NSC core–shell fibers were successfully fabricated with a coaxial nozzle. SCs in the shell portion were demonstrated to express higher abundances of neurotrophic factor genes than in petri dishes. Enhanced overall cell proliferation and differentiation tendency of NSCs were observed in this co-culture model, which could be due to their nourishment by secreted neurotrophic factors. This model may have strong potential in SCI repair.

## Funding

This work was supported by Chinese army open Grant [No. BWS17J036] and ‘Biomanufacturing and Engineering Living Systems’ Overseas Expertise Introduction Center for Discipline Innovation [No. G2017002].

*Conflict of interest statement*. None declared.
